# Effects of faba beans with different concentrations of vicine and convicine on egg production, egg quality and red blood cells in laying hens

**DOI:** 10.1017/S1751731116002688

**Published:** 2016-12-29

**Authors:** M. Lessire, V. Gallo, M. Prato, O. Akide-Ndunge, G. Mandili, P. Marget, P. Arese, G. Duc

**Affiliations:** 1 Institut National de la Recherche Agronomique (INRA), UR83 Recherches Avicoles, 37380 Nouzilly, France; 2 Department of Oncology, University of Torino, Via Santena 5 bis, 10126 Torino, Italy; 3 INRA, UMR1347 Agroécologie, BP 86510, 21000 Dijon, France

**Keywords:** faba bean, vicine, convicine, laying hen performances, red blood cells

## Abstract

The faba bean (*Vicia faba* L.) is a potential source of proteins for poultry, mainly for laying hens whose protein requirements are lower than those of other birds such as growing broilers and turkeys. However, this feedstuff contains anti-nutritional factors, that is, vicine (V) and convicine (C) that are already known to reduce laying hen performance. The aim of the experiment reported here was to evaluate the effects of a wide range of dietary V and C concentrations in laying hens. Two trials were performed with laying hens fed diets including 20% or 25% of faba bean genotypes highly contrasting in V+C content. In Trial 1, faba beans from two tannin-containing cultivars, but with high or low V+C content were dehulled in order to eliminate the tannin effect. In addition to the contrasting levels of V+C in the two cultivars, two intermediate levels of V+C were obtained by mixing the two cultivars (70/30 and 30/70). In Trial 2, two isogenic zero-tannin faba bean genotypes with high or low V+C content were used. In both trials, a classical corn–soybean diet was also offered to control hens. Each experimental diet was given to 48 laying hens for 140 (Trial 1) or 89 (Trial 2) days. Laying performance and egg quality were measured. The redox sensitivity of red blood cells (RBCs) was assessed by measuring hemolysis and reduced glutathione (GSH) concentration in these cells. Egg weight was significantly reduced by the diets containing the highest concentrations of V+C (*P*<0.0001) in Trial 1 and slightly reduced (*P*<0.10) in Trial 2, but only weak linear relationships between egg weight and dietary V+C concentration were established. No negative effect of V+C level was observed for egg quality parameters. In contrast, certain parameters (i.e. Haugh units, yolk color) were improved by feeding low V+C diets *(P*<*0.05).* Hemolysis of RBCs was higher in hens fed high V+C diets. A decrease in GSH concentration in RBCs of hens fed the highest levels of V+C was observed. Faba bean genotypes with low concentrations of V+C can therefore be used in laying hen diets up to 25% without any detrimental effects on performance levels or egg characteristics, without any risk of hemolysis of RBCs.

## Implications

In human nutrition, vicine (V) and convicine (C) from the faba bean are potentially harmful oxidants that may destroy red blood cells (RBCs), leading to anemia. The negative effects of these compounds have been demonstrated in poultry nutrition, but relationships between reduction of laying hen performance and RBC integrity have not been fully studied. As new faba bean genotypes with reduced amounts of V and C are now available, it has become possible to improve the characterization of the relationships between dietary V and C concentrations and the responses of laying hens. The results of this study should make it possible to define the maximum incorporation rate of the faba bean in the diets of laying hens according to its V and C concentration.

## Introduction

The seeds of the faba bean plant (*Vicia faba* L.) have been shown to be good sources of protein in animal and human nutrition. However, it has been established that some seed components such as condensed tannins, V and C reduce their nutritional value in non-ruminant animals (Crépon *et al*., 2010). Tannins are polyphenolic compounds concentrated in the seed coat (5 to 10 g/kg seed dry matter (DM)) and there is agreement in the literature (Crépon *et al*., 2010) that they reduce protein digestibility in pigs and poultry. Zero tannin faba bean varieties have been developed by genetic selection (Grosjean *et al*., 1995; Crépon *et al*., 2010). Vicine and convicine are glucopyranosides contained in the cotyledons of fresh and mature seeds of the faba bean. They are inactive *per se*, but are hydrolyzed by endogenous *β*-glucosidase of the faba bean or by the intestinal microflora of the animal into the powerful redox-active derivatives divicine (D) and isouramil (I), respectively, (Arese and De Flora, 1990) which can enter the blood compartment.

Wide genetic variability has been discovered for V and C content in the faba bean seed under the control of the major *VC* gene (Duc *et al*., 1999). In conventional cultivars carrying the *vc*+ allele, the V+C content ranges from 6 to 14 g/kg DM, whereas homozygous genotypes for the *vc*− allele have 10 to 20-fold reduced content of both V and C (Crépon *et al*., 2010). Selection seems to be a more efficient way of reducing the V and C content compared with thermal treatments (Cardador-Martinez *et al*., 2012). It has been shown that tannins and V+C reduce metabolizable energy (ME) values in adult cockerels and broilers (Grosjean *et al*., 2000; Metayer *et al*., 2003), but the effects of tannin and/or V+C could not be distinguished from each other in most experiments. Vilarino *et al.* (2009) demonstrated the additivity of the negative effects of tannins and V+C on ME in broiler chickens. No differences in nutrient content of the seed have been observed according to their V and C concentration (Duc *et al*., 1999) that might explain differences in ME values, and no clear effect of V+C on nutrient digestibility has been quantified in poultry. Other studies have established that reduction of egg production in laying hens depends on the dietary concentration of V+C (Vogt 1972; Guillaume and Bellec, 1977; Fru-Nji *et al*., 2007). Incorporation of faba bean extracts containing V+C in laying hen diets has demonstrated the negative effects of these compounds on egg production and egg size (Muduuli *et al*., 1981; Olaboro *et al*., 1981).

Unfortunately, cultivation of the improved cultivars with low V and C concentrations in Europe is low; for example they represented only 13% of French faba bean production in 2014 (Carrouée, personal communication).

In human nutrition, high levels of V+C present in commercial faba beans are potentially dangerous to individuals with low-activity variants of the glucose-6-phosphate dehydrogenase enzyme (G6PD). Divicine and Isouramil, the redox-active derivatives of V and C, oxidize reduced glutathione (GSH) in RBCs. GSH is a powerful scavenger of oxidant radicals, thus protecting the integrity of RBCs, whereas G6PD keeps glutathione in the reduced form. Ingestion of the faba bean by G6PD-deficient human subjects oxidizes GSH irreversibly and may damage RBCs, leading to a severe anemia syndrome called favism (Arese and De Flora, 1990). In laying hen, it has been shown that a vicine extract from faba bean, corresponding to diets containing 40% to 80% faba bean, increased erythrocyte hemolysis (Muduuli *et al*., 1981).

The aim of the study reported here was to determine the effects of V and C on the egg production and RBC integrity of laying hens fed diets including tannin-free faba beans by using genotypes with different levels of V and C. Two trials were performed with laying hens. In Trial 1, hens were fed diets containing 20% of conventional dehulled (in order to suppress tannins) faba beans containing a wide range of V+C; in Trial 2, hens received diets containing 25% of two experimental isogenic zero tannin genotypes of faba beans with very high or very low levels of V+C. As RBCs are target cells to evaluate the pro-oxidant effect of faba bean ingestion, their sensitivity to oxidant damage was analyzed in birds fed with the experimental diets.

## Material and methods

Experimental procedures and animal care were carried out according to current French legislation and under authorization granted to Lessire (N° 006 495) by the French Ministry of Agriculture.

### Trial 1

#### Faba bean characteristics and diets

Seeds of two spring faba bean plants, with high (seed variety Marcel) and low (seed variety Divine) V+C concentrations were produced in the field at INRA, Dijon, France. These two genotypes have tannin-rich seeds. As tannins are concentrated in the seed coats (Crépon *et al*., 2010), the two faba bean batches were mechanically dehulled by the method used for the preparation of soybeans before solvent extraction (Moore, 1983). The method includes a cracking step followed by a separation step. Cracking was performed with corrugated rollers resulting in beans being broken to a quarter/sixth of their initial size. Separation of the hulls from the cotyledons was carried out in an apparatus where the particles were graded according to size and the hulls aspirated. These operations were performed at the CREOL pilot plant (Pessac, France) with a Damann Croes cracking mill and a DENIS D50 ‘cleaner separator.’

Only the cotyledon fraction of the beans was included in the experimental diets. The control diet (A) was mainly composed of corn and soybean meal. The experimental diets (Table 1) contained 200 g/kg of one of the two faba bean varieties (diet B=cv. Marcel, diet E=cv. Divine) or a mixture of both (diet C=70 cv. Marcel/30 cv. Divine or diet D=30 cv. Marcel/70 cv. Divine) in order to obtain a gradation of V+C concentrations (Table 2). All diets were calculated to be isocaloric using an estimate of 12.14 MJ apparent metabolizable energy(AME)/kg (as-fed) for dehulled faba beans. This value was calculated using the value given by Sauvant *et al*. (2004) for raw white flowered beans and the difference in AME value obtained by Nalle *et al*. (2010) between raw and dehulled faba beans (1.6 MJ/kg DM). The diets were also calculated to be isonitrogenous using analyzed values for the protein content. Amino acid composition was calculated using the analyzed N content of the faba beans tested and the amino acid profile according to Sauvant *et al*. (2004). The composition and calculated characteristics of the experimental diets are given in Table 1. All diets were given as mash.

**Table 1:** Composition and calculated characteristics (as-fed) of the experimental diets, Trials 1 and 2

	Trial 1			Trial 2		
Ingredients (g/kg)	Diet A (control)	Diet B (high V+C)	Diet E (low V+C)	Diet A (control)	Diet B (high V+C)	Diet C (low V+C)
Faba bean		200.00	200.00		250.00	250.00
Maize	619.00	543.60	523.20	618.20	492.00	477.53
Corn gluten meal	42.42	47.20	48.40	10.00	7.24	17.00
Soybean meal	196.60	74.30	72.00	240.50	109.0	113.60
Wheat			21.20			
Rapeseed oil	16.70	8.30	8.60	20.15	30.00	30.00
Lucerne meal	15.00	15.00	15.00			
dl-methionine	1.36	1.80	1.80	1.55	2.15	2.05
l-lysine-HCl	0.62	0.50	0.60	0.94	0.11	0.43
l-tryptophan	0.20	0.50	0.50	0.16	0.43	0.44
l-threonine		0.40	0.40		0.12	0.13
Salt	3.00	3.00	3.00	3.00	3.00	3.00
Dicalcium phosphate	11.80	11.40	11.40	11.88	11.90	11.80
Limestone	38.30	39.00	39.00	38.50	38.90	38.90
Sea shells (Calcicoque)	50.00	50.00	50.00	50.00	50.00	50.00
Trace elements^1^	5.00	5.00	5.00	5.12	5.15	5.12
Calculated characteristics (%, as-fed)						
AME (MJ/kg)	11.51	11.51	11.51	11.51	11.51	11.51
CP	17.0	17.0	17.0	17.1	17.1	17.1
Lysine	0.80	0.80	0.80	0.90	0.90	0.90
Methionine	0.43	0.43	0.43	0.43	0.43	0.43
Tryptophan	0.18	0.18	0.18	0.19	0.19	0.19
Calcium	3.8	3.8	3.8	3.8	3.8	3.8
Available P.	0.33	0.33	0.33	0.33	0.33	0.33

V=vicine; C=convicine; AME=apparent metabolizable energy.

1 Trace elements: trace elements mix which supplied per kilogram of diet : Co, 0.6 mg; Cu, 20 mg; Fe, 58 mg; I, 2 mg; Mn, 80 mg; Se, 0.2 mg; Zn, 90 mg; retinyl acetate, 15 000 IU; cholecalciferol, 4300 IU; dl-alpha tocopheryl acetate, 100 mg; thiamine mononitrate, 5 mg; riboflavin, 8 mg; calcium pantothenate, 25 mg; cyanocobalamin, 0.02 mg; menadione, 5 mg; pyridoxine hydrochloride, 7 mg; folic acid, 3 mg; biotin, 0.3 mg; niacin, 100 mg; choline chloride, 550 mg; antioxidant (buthylhydroxyanisole, propyl gallat, ethoxyquin), 50 mg and red pigment (Trial 2).

**Table 2:** Analyzed composition (dry matter (DM) basis) of faba bean genotypes and diets in trials 1 and 2

Faba bean	Diet type	CP (%)	V (g/kg)	C (g/kg)	V+C (g/kg)	AME (MJ/kg)
Marcel (high V+C)		33.7	3.42	2.43	5.85	
Divine (low V+C)		33.7	0.48	0.21	0.69	
	Trial 1-diet A	18.1	0	0	0	12.90^a^
	Trial 1-diet B	18.4	0.68^1^	0.49^1^	1.17^1^	12.64^b^
	Trial 1-diet C	18.4	0.51^1^	0.35^1^	0.86^1^	
	Trial 1-diet D	18.4	0.27^1^	0.17^1^	0.45^1^	
	Trial 1-diet E	18.4	0.10^1^	0.04^1^	0.14^1^	12.36^c^
EE0TV (high V+C)		32.9	4.69	2.40	7.09	
EE0T0V (low V+C)		29.8	0.36	0.04	0.40	
	Trial 2-diet A	19.2	0	0	0	12.67^a^
	Trial 2-diet B	19.4	1.17^1^	0.60^1^	1.77^1^	12.65^a^
	Trial 2-diet C	19.5	0.09^1^	0.010^1^	0.10^1^	12.77^a^

V=vicine; C=convicine; AME=apparent metabolizable energy. ^a,b,c^In each trial, values with different superscript letters differ significantly (*P*<0.05).

1 Calculated values according to the incorporation rate of faba beans and their V and C concentrations.

#### Feeding experiments

In total, 240 18-week-old laying hens (ISA Brown) were used. They were kept in individual cages (250×460×440 mm) and fed a standard diet *ad libitum*. During 3 weeks they were checked daily. Hens with low feed consumption or egg production were replaced by others birds which were from the same delivery, fed with the same standard diet and housed in the same cage facility. When they were 21 weeks of age, they received one of the five experimental diets (Table 1). Six replicates were allocated to each experimental diet, each replicate comprising a group of eight contiguous cages sharing a common feeder. The daily quantity of feed was provided every morning to prevent spillage; feed consumption was calculated per replicate. The experiment was begun 1 week after providing the experimental diets. The experiment was conducted over 140 days, divided into five periods of 28 days. Hens were weighed at the beginning and at the end of the experiment, and weight gain was calculated. The number of eggs was checked daily for each hen, and all the eggs were weighed daily. Eggs were classified as saleable or non-saleable (double, dirty, broken). Mean egg weight was calculated on ‘saleable’ eggs; egg mass was calculated as the total weight produced, including the weights of non-saleable eggs. The feed conversion ratio (FCR) was calculated per period by dividing feed consumption by egg mass produced in each replicate, and the same calculation was performed to obtain the FCR of the entire experimental period. At the end of each 28-day period, one egg per hen was collected to measure egg and shell weights. The egg shell index was calculated (Sauveur, 1988), and breaking strength (Instron 5543, Instron, Guyancourt, France), and yolk color (Miniscan TM, Hunterlab, Noisy le Grand, France) were measured using the CIELAB system, diameter and height of the yolk, and height of the egg white (albumen density) that is Haugh units (Sauveur, 1988) were determined. The number of blood spots was also determined for each egg.

### Trial 2

#### Faba bean characteristics and diets

Two isogenic zero-tannin genotypes of faba beans differing only in *VC* gene alleles selected by INRA Dijon were produced in the field (Duc *et al*., 1999): EE0TV (zero-tannin, rich in V and C) and EE0T0V (zero tannin, low in V and C). Two experimental diets containing 25% faba beans (diet B=25% EE0TV, diet C=25% EE0T0V) were compared with a classical maize/soybean meal (diet A). The diets were calculated to present the same nutritional values. The estimated AME value of the two faba beans varieties was 10.61 MJ/kg as-fed basis. As in Trial 1, protein content was measured and amino acid content was estimated. The composition of the experimental diets is reported in Table 1. Diets were given as mash.

#### Feeding experiments

In all, 144 18-week-old laying hens (ISA Brown) were used. Hens were kept in cages identical to those used for Trial 1. During 2 weeks they were checked daily. Hens with low feed consumption or egg production were replaced by others birds which were from the same delivery, fed with the same standard diet and housed in the same cage facility. Six replicates of eight hens were allocated to each experimental diet. Hens were 20 weeks of age when they received the experimental diets and measurements started 1 week later. The duration of the trial was 112 days and was divided into four periods of 28 days. Measurements were the same as for Trial 1. Eggs were also classified as saleable or non-saleable.

### Trials 1 and 2

#### Analysis of faba bean seeds and diets

Faba beans were analyzed for CP levels (N×6.25) according to Association of Official Analytical Chemists (2004). Vicine and Convicine levels were analyzed by HPLC (Quemener *et al*., 1982). All the experimental diets were checked for protein content. AME values of control diets and those containing Marcel, Divine, EE0TV and EETOV faba beans, (i.e. diets A, B and E in Trial 1 and diets A, B and C in Trial 2) were determined in eight adult roosters/diet fed *ad libitum* according to Lessire (1990).

#### Red blood cell studies: redox sensitivity of red blood cells

All reagents were from Sigma-Aldrich, St. Louis, MO, unless otherwise stated. Redox sensitivity of RBCs was assessed by measuring RBC hemolysis in hens fed control diet or diets containing different percentages of faba bean with high (Marcel or EE0TV beans) or low (Divine or EE0T0V beans) levels of V+C. Hemolysis of RBCs was analyzed as described by Stagsted and Young (2002). The results were expressed as percentage of hemolysis using the absorbance value at 406 nm of RBCs lysed by addition of Triton X-100.

#### Assay of reduced glutathione levels in red blood cells

GSH levels in the RBCs of laying hens were assayed as described by Beutler (1975) and expressed as mmoles/ml whole RBC.

### Statistical analysis

Production parameters for each hen were calculated for each 28-day period, then a mean was calculated for the whole trial duration. Daily feed consumption by hens was calculated by dividing the consumption of each group of hens by the number of hens (eight) and by the duration of the period (28 days), then a mean was used for the whole trial duration. The same calculation was made for egg quality measurements. Statistical analysis was performed using the Statview statistical program (SAS Institute, Cary, NC, USA). The results were analyzed by one factor (the diet) ANOVA, and a Fisher’s test was used to compare the means. *P*<0.05 was considered to be significant. Relationships between levels of V+C and performances of the birds were tested by regression.

## Results

### Trial 1

#### Faba bean and diet composition, and hen performance levels

The composition of the two faba bean cultivars utilized in Trial 1 is presented in Table 2. Their protein content was similar, amounting to 33.7%, DM basis. As expected, Marcel beans had higher levels of V and C compared with Divine beans. No differences in the protein content of the experimental diets were detected, and the analyzed values (18.1% to 18.4% DM basis) were close to the target value: 17% as-fed, that is 18.5% to 18.8% DM basis. The target AME value of the experimental diets (11.51 MJ/kg as-fed, i.e. 12.73 MJ/kg DM basis) was slightly lower than the value determined for the control diet A (12.90 MJ/kg DM). By contrast, the determined value was lower for diet E with 20% Divine (12.36 MJ/kg DM). Diet B with 20% Marcel was intermediate (12.64 MJ/kg DM) and very close to the target value. Such results indicate overestimation of the AME values of the two faba beans, mainly for Divine.

Only two hens were discarded during the experiment, one from diet B and one from diet D. Cumulative performance levels of the hens over the 140-day trial are presented in Table 3. Weight gain, laying rate, number of eggs and FCR were not affected by the composition of the feed. By contrast, feed consumption was significantly increased by diets containing Divine faba beans, but no significant relation with V+C content was detected. Mean egg weight and egg mass were reduced by feeding the hens with diet B containing 20% Marcel. Correlations were calculated between birds’ performances and V, C and V+C dietary concentrations and determined dietary AME values. By construction V, C and V+C concentrations are correlated, they are also correlated with diet AME value as the lower ME value is observed with Divine diet. Weak linear relationships between egg production and V+C dietary contents (mg/kg) were obtained:

∙ Egg mass (g)=8441.4−0.44 (V+C), *R* ^2^=0.09 with *P*<0.0001.
∙ Egg weight (g)=58.2−0.003 (V+C), *R* ^2^=0.12 with *P*<0.0001.

**Table 3:** Effects of vicine (V) and convicine (C) on hen performance levels and quality of eggs over the 140-day experimental period, trial 1

	Diet A (control)	Diet B (Marcel, high V+C)	Diet C (2/3 Marcel+1/3 Divine)	Diet D (1/3 Marcel+2/3 Divine)	Diet E (Divine, low V+C)	SEM	*P*-value
Weight gain (g)	562	514	490	532	504	10.0	0.18
Feed consumption (g/day)	109.5^b^	108.7^b^	111.2^ab^	113.6^a^	114.9^a^	0.71	0.01
Laying rate (%)	96.9	97.2	96.3	97.7	96.9	0.27	0.54
Mean egg weight^1^ (g)	56.6^b^	54.3^c^	56.3^b^	56.3^b^	57.9^a^	0.22	<0.0001
Total number of eggs^2^	135.7	136.0	134.7	136.6	135.6	0.37	0.58
Egg mass^2^ (g)	8183^a^	7868^b^	8148^a^	8197^a^	8382^a^	36.7	0.0003
Feed conversion ratio	1.873	1.927	1.911	1.930	1.920	0.008	0.11
Eggshell breaking strength (N)	37.1^b^	38.4^ab^	39.5^a^	37.7^b^	37.9^b^	0.27	0.05
Eggshell index	8.53	8.51	8.65	8.42	8.51	0.03	0.20
Haugh units	80.6^d^	86.3^ab^	86.6^a^	83.9^bc^	82.1^cd^	0.44	<0.0001
Yolk color							
Luminance L*	52.6	52.2	52.1	52.2	52.6	0.09	0.26
Redness a*	7.38^b^	7.90^a^	7.82^a^	7.83^a^	7.88^a^	0.04	<0.0001
Yellowness b*	49.7	49.9	50.0	50.3	50.6	0.14	0.36
Number of blood spots	1.12	0.98	0.97	1.05	1.26	0.05	0.35
Ratio diameter/height of yolk	2.58^c^	2.65^a^	2.64^ab^	2.63^abc^	2.59^bc^	0.008	0.03

^a,b,c^Values within a row with different superscript letters differ significantly (*P*<0.05).

1 Calculated on saleable eggs.

2 Including saleable and non-saleable eggs (broken, dirty, etc.).

No significant quadratic effect was detected.

The effects of the experimental diets on the quality of eggs are presented in Table 3. Breaking strength of eggs from hens fed the faba bean diets were at least equal to those from hens fed the corn–soybean diet, it was slightly improved in the lightest eggs coming from hens fed the highest V+C diets. Haugh units and the diameter/height ratio of the yolk were significantly improved by feeding the hens with faba beans. Yolk color was not noticeably modified, whereas redness (a*) was improved by inclusion of faba beans in the diet. In contrast, the egg shell index, which depends on shell weight and egg weight, and the number of blood spots were not different between diets.

#### Redox sensitivity of red blood cells

**Figure 1:**
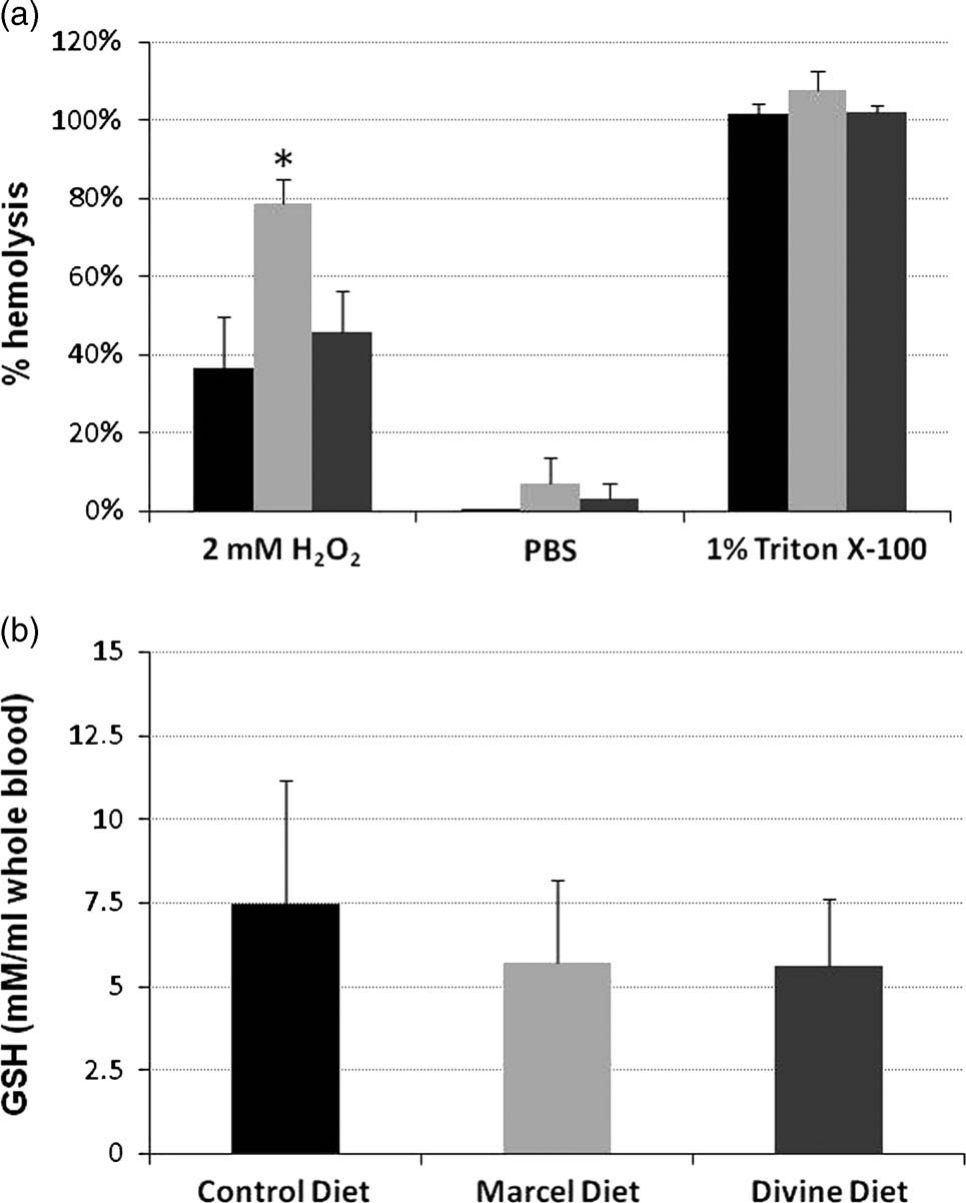
Screening assay for red blood cells (RBC) redox sensitivity (a) and measurement of Glutathione (GSH) levels in RBCs (b) from hens of trial 1. (a) Hen RBCs were incubated on microplate shaker (1000 r.p.m.) for 12 h in the dark at room temperature in the presence of 2 mM H_2_O_2_ after 2 weeks of control soybean diet (black bars), or a diet supplemented with 20% high vicine (V)+convicine (C) faba beans (Marcel beans, light gray bars) or a diet supplemented with 20% low V+C faba beans (Divine beans, dark gray bars). The same RBCs were incubated for 12 h under the same conditions: as a negative control in phosphate-buffered saline (PBS) only, or as a positive control with 100% hemolysis after addition of 1% Triton X-100. Column data represent mean values±SD of four independent experiments analyzed for statistical significance by two-sided Student’s *t*-test. Percentage of hemolysis in RBCs from hens fed the high V+C diet was significantly higher than in control RBCs (*P<0.002) and those from hens fed the low V+C diet (*P<0.003). (b) GSH levels (expressed as mmoles/ml whole blood) were measured in hen RBCs after 1 week of control, Marcel (high V+C) or Divine (low V+C) diet. For details, see (a) and Materials and Methods section.

Redox sensitivity was assessed by measuring H_2_O_2_-induced hemolysis of RBCs from hens after 2 weeks of feeding the control diet or diets containing 20% faba beans either with high (Marcel diet) or low (Divine diet) V+C concentration. RBCs were incubated for 48 h and analyzed time-dependently after 1, 2, 4, 12, 24 and 48 h incubation, with or without 0.02, 0.2 and 2 mM H_2_O_2_, a powerful oxidant and hemolytic agent. As shown in Figure 1a, the percentage of hemolysis in RBCs from hens fed the high V+C Marcel diet was significantly higher compared with control RBCs (*P*<0.002) and to RBCs from hens fed the low V+C Divine diet (*P*<0.003). Hemolysis in RBCs from hens fed the low V+C diet was not significantly different from that of control RBCs. Negative controls (RBCs incubated in phosphate-buffered saline) and positive controls (RBCs incubated in 1% Triton X-100) showed no significant differences between the three diets.

#### Measurement of reduced glutathione levels in red blood cell

GSH levels were not significantly lower in the RBCs from laying hens fed a high V+C diet (Figure 1b), and tended to be lower at other time points without reaching constant significance compared with control values (not shown).

### Trial 2

#### Faba bean and diet composition, and hen performance levels

Protein content of EE0TV (32.9% DM) was higher than that observed for EE0T0V (29.8% DM) (Table 2). As expected, V and C levels were strikingly higher in EE0TV than in EE0T0V, the V+C level in EE0TV being 18 times higher than in EE0T0V. The determined AME values of the three experimental diets were very similar (12.67, 12.65 and 12.76 MJ/kg DM for diets A, B and C, respectively) and differences were not significant (*P*=0.66). These determined values were in the range of the calculated values: 11.30 MJ as-fed, that is 12.69 MJ/kg DM. The protein levels of the experimental diets were 19.2%, 19.4% and 19.5% (DM basis) for diets A, B and C, respectively. These values were consistent with the calculated value, namely 17.1% as-fed, that is, 19.2% DM.

The duration of the experiment was scheduled to last 112 days, but due to lack of feed it lasted only 89 days. As shown in Table 4, production performance parameters were independent of the diet. Only significant differences were observed between high and low V+C diets for Haugh units, redness (a*) of the yolk and the diameter/height ratio of the yolk.

**Table 4:** Effects of vicine (V) and convicine (C) on hen performance levels and egg quality over the 89-day period, trial 2

	Diet A (control)	Diet B (EE0TV, high V+C)	Diet C (EE0T0V, low V+C)	SEM	*P*-value
Weight gain (g)	257	320	274	10.8	0.07
Feed consumption (g/d)	106.2	107.2	107.7	1.03	0.85
Laying rate (%)	98.5	98.0	98.2	0.26	0.72
Mean egg weight^1^ (g)	62.5	60.4	61.4	0.32	0.10
Total number of eggs/hen^2^	86.7	86.2	86.4	0.23	0.63
Egg mass^2^ (g/hen)	5410	5246	5346	31.6	0.10
Feed conversion ratio	1.780	1.819	1.792	0.050	0.40
Eggshell breaking strength (N)	36.1^b^	38.3^a^	35.9^b^	0.45	0.04
Eggshell index	8.56	8.41	8.46	0.04	0.40
Haugh units	86.6^b^	92.6^a^	87.4^b^	0.59	<0.0001
Yolk color					
Luminance L*	53.2	53.1	53.3	0.14	0.68
Redness a*	5.59^b^	5.56^b^	5.85^a^	0.05	0.04
Yellowness b*	48.1^b^	48.5^ab^	49.2^a^	2.19	0.05
Blood spots number	0.70	0.48	0.52	0.05	0.11
Ratio diameter/height of the yolk	2.60^b^	2.69^a^	2.59^b^	0.011	0.0004

^a,b^Values within a row with different superscript letters differ significantly (*P*<0.05).

1 Calculated on saleable eggs.

2 Including saleable and non-saleable eggs (broken, dirty, etc.).

#### Redox sensitivity of red blood cells

**Figure 2:**
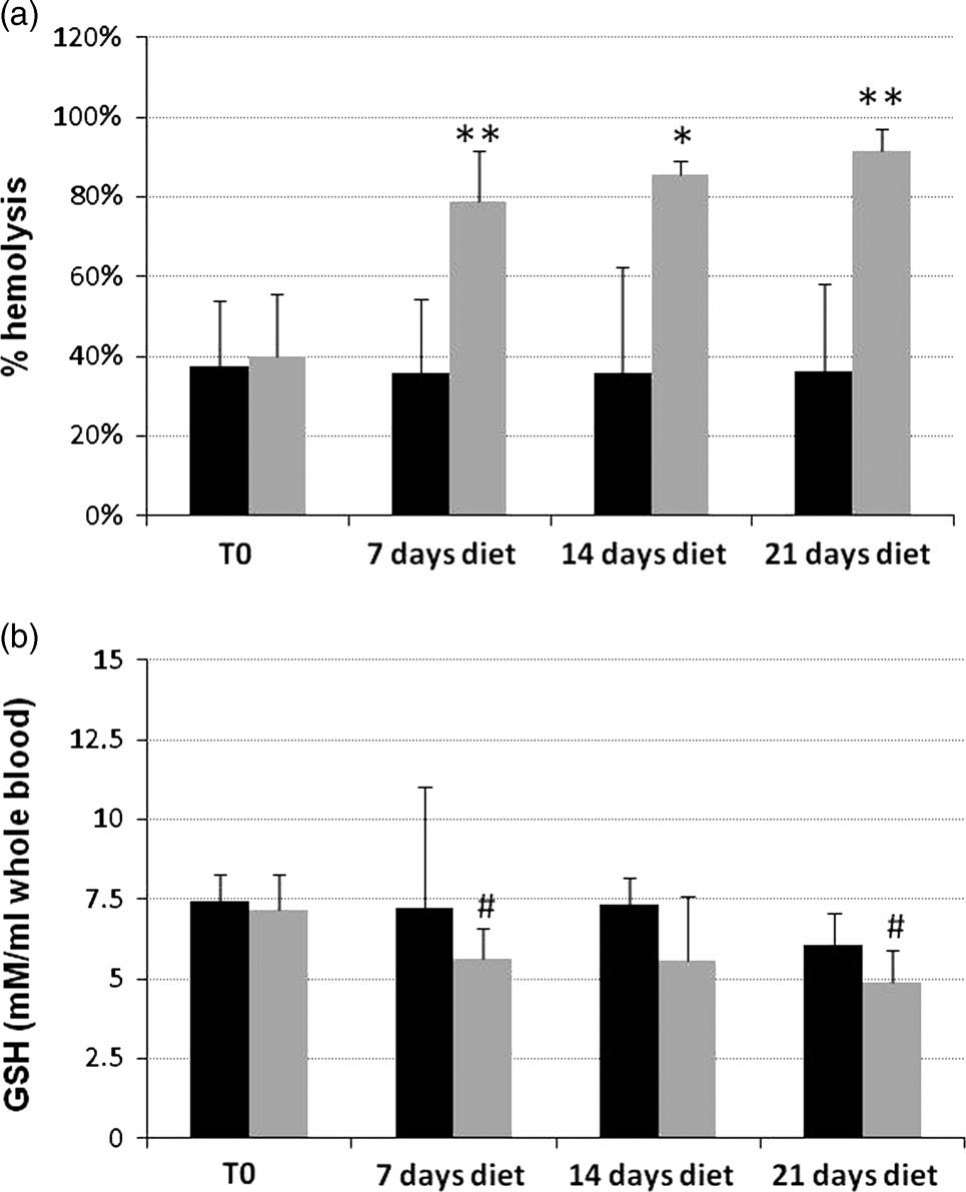
Screening assay for red blood cells (RBC) redox sensitivity (a) and measurement of Glutathione (GSH) levels in RBCs (b) from hens in Trial 2. (a) Hen RBCs were incubated on a microplate shaker (1000 r.p.m.) for 12 h in the dark at room temperature, in the presence of 2 mM H_2_O_2_ after 3 weeks of control soybean diet (black bars) or a diet supplemented with 25% high vicine (V)+convicine (C) faba beans (light gray bars) (check points on days 0, 7, 14 and 21). Column data represent mean values±SD of four independent experiments analyzed for statistical significance by two-sided Student’s *t*-test. Percentage of hemolysis in RBCs from hens fed the high V+C diet was significantly higher than control RBCs (all **P<0.003, but *P<0.01 for day 14), and then at time 0 for the control diet and time 0 for the faba bean diet (*P*<0.001). (b) GSH levels (expressed as mmoles/ml whole blood) were measured in hen RBCs after 3 weeks of control soybean diet (black bars) and a diet supplemented with 25% high V+C faba beans (light gray bars) (check points on days 0, 7, 14 and 21). Column data represent mean values±SD of four independent experiments, analyzed for statistical significance by two-sided Student’s *t*-test. The differences between days 7 and 21 *v.* day 0 were statistically significant (^#^ *P*<0.05).

Redox sensitivity was assessed by measuring H_2_O_2_-induced hemolysis of RBCs from hens fed the control diet and the diet containing 25% faba beans with high V+C concentration, in a time-course experiment. RBCs (check points on days 0, 7, 14 and 21) were incubated for 12 h in the presence of 2 ml H_2_O_2_, and hemolysis was measured. As shown in Figure 2a, the percentage of hemolysis was significantly higher in RBCs from V+C-fed hens than in control RBCs (all *P*<0.03, but *P*<0.01 for day 7), and than in control and faba bean diets (*P*<0.001) at point 0.

#### Measurement of reduced glutathione levels in red blood cells

As shown in Figure 2a, GSH was measured in RBCs in a 3-week time course experiment (check points on days 0, 7, 14 and 21). The results showed a decrease in GSH concentration in the RBCs from hens fed the high V+C diet on days 7, 14 and 21 in relation to day 0 (Figure 2b), whereas GSH concentration in the RBCs from hens fed the control soybean diet was fairly stable. Despite within-sample variability, the differences between days 7 and 21 and day 0 were statistically significant (*P*<0.05).

## Discussion

During the two trials in this study, laying hens were fed diets containing 20 (Trial 1) and 25% (Trial 2) faba beans with different levels of V and C. A wide range of dietary V and C concentrations was tested, V+C content varying from 0.10 to 1.77 g/kg. These levels were consistent with those used in previous studies performed in practical conditions to test faba bean inclusion in layer diets (Perez-Maldonado *et al*., 1999; Fru-Nji *et al*., 2007), but were by far lower than those tested by Muduuli *et al*. (1981) who used a 0.5% and 1% vicine preparation. In our both trials, mean egg weight was reduced by almost 2 g when laying hens were fed high V+C diets. This reduction was significant in Trial 1, which lasted for 140 days, but not in Trial 2 (*P*=0.10). This could be explained by the shorter duration of the latter (89 days) as we observed (data not presented) that the difference between the control diet and the highest V+C diet was 1.6 g at the end of the first 28-day period and reached a plateau (2.6 to 3 g) during the third one (Trial 1). A difference of 3.1 g of weight of egg between control diet and EE0TV diet was only observed at the end of trial 2. Egg mass followed the same trend. These results suggest a specific effect of V+C on egg composition: for example a reduction in the yolk fraction due to changes in lipid metabolism as previously observed by Fru-Nji *et al*. (2007) and Muduuli *et al*. (1981). By contrast, the positive effect of feeding the birds with high V+C diets on egg breaking strength could be explained by the reduction of egg weight, smaller is the egg higher is the breaking strength, as demonstrated previously (Sekeroglu and Altuntas, 2008). Other parameters such as feed consumption, FCR and number of eggs were not negatively affected by the experimental diets and other parameters such as Haugh units, color and the diameter/height ratio of the yolk were improved by reduced V+C content. These results are in agreement with previous reports (Perez-Maldonado *et al*., 1999; Fru-Nji *et al*., 2007). Improvement of Haugh units and the diameter/height ratio of the yolks of hens fed a faba bean diet could be explained by viscosity of the albumen. Changes in yolk color depend on the consumption of pigmentation substances (carotenoids) which come from the feed. In the present trials, the redness value (a*) of the yolk was modified by feeding the birds with faba bean rich diets. As the carotenoid concentrations of the diets were not determined, it was not possible to assume whether the differences observed came from the carotenoid concentration in the faba bean or from the other feedstuffs used (i.e. equilibrium between the dietary ingredients which contain carotenoids). The higher feed consumption of the hens fed diets C, D and E which contained faba bean cv. Divine (Trial 1) could be explained by the lower ME value of the diets due to an overestimate value of the dehulled Divine bean. This overestimate could be due to a lower dehulling effect than that used for the calculation of the AME value of Divine, as this bean does not contain tannins. For other studies, it would be necessary to measure ME value and amino acid digestibility of the beans before computing the diets.

The redox sensitivity of RBCs was assessed by measuring RBC hemolysis in hens fed the control diet and diets containing faba beans. After 2 weeks of feeding (Trial 1), the results indicated a clear increase in oxidant sensitivity and significantly higher percentage of hemolysis in RBCs from hens fed the Marcel bean diet (high V+C) compared with control RBCs and to RBCs from hens fed the Divine (low V+C) diet. GSH levels in the RBCs were not significantly lower in laying hens fed a high V+C diet and only tended to be lower at other time points than control values, without reaching significance. In Trial 2 redox sensitivity was analyzed time-dependently after 7, 14, and 21 days’ feeding a high or low V+C diet. A significant difference was observed after oxidative stress exerted by H_2_O_2_ treatment: low V+C samples were indistinguishable from controls, whereas high V+C samples were significantly more prone to oxidation and had significantly higher percentages of hemolysis. GSH levels were decreased in RBCs from hens fed the high V+C diet on days 7, 14 and 21, whereas GSH levels were fairly stable with the soybean diet. Faba bean components V and C (after activation in the host’s gut to redox-active divicine (D) and isouramil (I), respectively) are responsible for the antinutritional effects in FB-fed chicken. In human subjects lacking the enzyme G6PD (>400 000 million hemizygous males worldwide are affected), ingestion of raw FBs may elicit favism, a severe hemolysis due to the pro-oxidant activity of D and I. D and I in presence of GSH and hemoglobin in the host RBCs generate hydrogen peroxide and oxygen radicals at the expense of GSH and NADPH; the latter is oxidized to regenerate GSH. In the process a vast number of cellular targets, mostly proteins and lipids, are modified/damaged (Arese and De Flora, 1990). The final effects are profound modifications of target cells. For example, RBCs are destroyed leading to severe anemia. In chicken fed raw FBs, V and C activated in the gut are rapidly absorbed into the blood and transported by the circulation to all organs. Frohlich and Marquardt (1983) have measured the kinetics of V/I distribution in chicken fed with raw beans, showing rapidly reached high levels in organs such as liver and kidney. In the two trials we performed, we have selected RBCs as target cells to evaluate time-dependently the pro-oxidant effects of FB ingestion. RBCs were selected due to their easy accessibility to repeated testing and their sensitivity to oxidant damage. Moreover, it has been shown that chicken RBCs are orders of magnitude more sensitive to oxidative stress due to their low catalase activity. As D and I easily permeate into all cells, it may be assumed that egg-producing organs are also target of V/I and functionally disturbed leading to lower egg weight, but the real mechanisms remain to describe.

In conclusion, RBC parameters related to oxidant stress, such as hemolysis after oxidant challenge, and the changes in GSH levels were sensitive indicators of physiological modifications induced by high levels of V+C in the diet. It was not clear whether these modifications were the only raison for the reduction in egg weight we observed. For example, one can evoke the ME value of the diets in Trial 1. The administration of faba beans with low levels of V+C do not affect performances and there was no adverse effects of feeding large amounts (200 to 250 g/kg of diet) of those faba bean to laying hens on performances and risk of oxidative RBC damage or frank hemolysis. The results of these trials support the use of the new faba bean cultivars with low V+C concentrations in laying hen feed.
